# Nifedipine Promotes the Proliferation and Migration of Breast Cancer Cells

**DOI:** 10.1371/journal.pone.0113649

**Published:** 2014-12-01

**Authors:** Dong-Qing Guo, Hao Zhang, Sheng-Jiang Tan, Yu-Chun Gu

**Affiliations:** 1 Molecular Pharmacology Laboratory, Institute of Molecular Medicine, Peking University, Beijing, China; 2 Department of Surgery, Huashan Hospital, Fudan University, Shanghai, China; 3 The Department of Haematology, University of Cambridge, Cambridge, United Kingdom; The First Affiliated Hospital with Nanjing Medical University, China

## Abstract

Nifedipine is widely used as a calcium channel blocker (CCB) to treat angina and hypertension,but it is controversial with respect the risk of stimulation of cancers. In this study, we demonstrated that nifedipine promoted the proliferation and migration of breast cancer cells both *invivo* and *invitro*. However, verapamil, another calcium channel blocker, didn’t exert the similar effects. Nifedipine and high concentration KCl failed to alter the [Ca^2+^]_i_ in MDA-MB-231 cells, suggesting that such nifedipine effect was not related with calcium channel. Moreover, nifedipine decreased miRNA-524-5p, resulting in the up-regulation of brain protein I3 (BRI3). Erk pathway was consequently activated and led to the proliferation and migration of breast cancer cells. Silencing BRI3 reversed the promoting effect of nifedipine on the breast cancer. In a summary, nifedipine stimulated the proliferation and migration of breast cancer cells via the axis of miRNA-524-5p-BRI3–Erk pathway independently of its calcium channel-blocking activity. Our findings highlight that nifedipine but not verapamil is conducive for breast cancer growth and metastasis, urging that the caution should be taken in clinic to prescribe nifedipine to women who suffering both hypertension and breast cancer, and hypertension with a tendency in breast cancers.

## Introduction

Breast cancer is one of the most prevalent human cancers worldwide and accounts for 26% of all cancers (excluding basal and squamous cell skin cancers) in women according to the Cancer Statistics of 2008 [1]. Men also suffer breast cancer commonly with poorer outcomes due to delays in diagnosis. Accumulated results have revealed that the genetic mutations and individual living environment together contribute to the incidence of the breast cancer [2]–[4].

Calcium channel blockers (CCBs) disrupting the movement of calcium through calcium channels, include dihydropyridine, phenylalkylamine and benzothiazepine. Three kinds of reagents display different molecular structures and bind separately with receptor sites located in or near the calcium channel [5]. Among them, nifedipine (also named Adalat, Nifediac, Nifedical or Procardia) is commonly used in the treatment of angina and hypertension.

Several epigenetic studies have implicated that CCBs might be involved in cancer stimulation, but the risk of CCBs is controversial [6]–[9]. CCBs facilitated the division of cells with malignant potential, thus they increased the risk of cancers especially the breast cancer [10]. In a prospective cohort study, 451 out of 5052 people aged about 71 years had taken CCBs for four years and the hazard ratio for cancer associated with CCBs compared with not taking CCBs was 1.72 [11]. Fitzpatrick and colleagues chose 3198 women aged over 65 years from 4 different places. They found that the elevated risk of breast carcinoma was associated with use of CCBs and the hazard ratio was 2.57 [12]. Additional studies consistently showed CCBs and certain diuretics increased the risk of breast carcinoma among older women [13]. Clinic observation on the complication of nifedipine commonly led to mental symptoms and male breast hypertrophy. However, there are other studies showing that CCBs had no relation with cancers [11], [14]–[16]. Besides these reports, amlodipine, diltiazem and verapamil were even found to inhibit the growth of breast cancer in themodel of nude mice [17] as well as the meningioma growth [18].

According to the statistics, about 45% of hypertension patients are women. Dihydropyridine e.g. nifedipine accounts for 1/10 of compounds which are daily used in the clinic treatment of hypertension and associated cardiac diseases. It is therefore critical to understand whether CCBs can promote breast cancers and what is the mechanism underlining this cancinoma provocation. In this study, we found and confirmed that nifedipine, but not verapamil, could promote breast cancer both *invivo* and *invitro*. Nifedipine decreased miRNA-524-5p, resulting in the up-regulation of brain protein I3 (BRI3). Erk pathway was consequently activated and led to the proliferation and migration of breast cancer cells. Silencing BRI3 reversed the promoting effect of nifedipine on the breast cancer.

## Materials and Methods

### Cell culture and reagents

MCF-7 and MDA-MB-231 cells were purchased from Cell Library of Chinese Academy of Sciences. MCF-7 were cultured in the DMEM medium(Promocell, Germany) with 10% FBS (GIBCO, US) and maintained at 37°C and in 5% CO_2_. MDA-MB-231 were grown in L-15 medium(Promocell, Germany) with 10% FBSand maintained at 37°C and in atmospheric air.

Nifedipine (Sigma), verapamil (Sigma), MTT (5 mg/ml, Sigma), Paraformaldehyde (4%, Solarbio), Crystal violet (0.1%, Solarbio), RIPA (Solarbio), PMSF (Solarbio), Cocktail (25x, Roche), Fura-8™ (AAT Bioquest), DMSO (Sigma), CMC-Na (Beijing Chemical Technology, China), Nifedipine Sustained-Release Tablets (Yangtze River Pharmaceutical Group, China), Verapamil Hydrochloride Tablets (Central Pharmaceutical Co., Ltd, China).

### Animal studies

Animal experiments conformed to the Guide for the Institutional Animal Care and Use Committee (IACUC) and was approved by the Ethics Review Board for Animal Studies of Institute of Molecular Medicine(IMM), Peking University (Permit Number: IMM-GuYC-1). Balb/c nude mice were purchased from Vital River (Beijing, China). Five to seven weeks old female nude mice were used for tumor injections. MDA-MB-231 cells were infected by PsinDest5.1-GFP (a gift from Professor YM Wang) to generate stable MDA-MB-231-GFP cells. 5×10^6^ MDA-MB-231-GFP breast cancer cells were suspended in 100ul cold D-PBS and injected into the second fat pad of nude mice described as the previous report [19]. One week after injection, when the breast tumor size reached 100mm^3^, nude mice were then fed with nifedipine (4.8 mg/kg) (Yangtze River Pharmaceutical Group), verapamil (4.8 mg/kg) (Central Pharmaceutical Co., Ltd, China) by gauging. CMC-Na (Beijing Chemical Technology) and H_2_O were used as controls. Nude mice were weighted every day and tumor size was measured every other day.

### *In vivo* imaging of tumor metastasis

Tumor metastatic was monitored using *invivo* fluorescence. Nude mice were anesthetized using isoflurane. The fluorescence was emitted from the tumors and detected using a Kodak Imaging Systems (Kodak, New Haven, CT). Images were digitally captured and overlaid onto the X-ray reference image (Kodak Imaging System).

### Proliferation assays

For determination of proliferation, MTT assay was used as reported previously. Briefly, breast cancer cells were seeded at a density of 1,000 per well into 96-well cell culture plates and allowed them to adhere for 24h. After incubation with various concentration of nifedipine for 48h, 20 µl of MTT was added to each well for 3h incubation. Subsequently, cells were dissolved by 150 µL of DMSO, mixed and measured the absorbance (A) by Multiskan (Thermo) at 540nm. The relative proliferation rate was calculated by the absorbance ratio of the nifedipine-treated group to the control group.

### Cell migration assays

For transwell migration assays, harvested cells (1×10^5^ cells) supplement with 100ul of serum-free medium were replated onto the upper chamber (a 6.5-mm polycarbonate membrane with 8.0-µm pores; Corning, NY) and the chamber was placed in complete medium with 10% FBS and nifedipine. After 24hours, cells were fixed with 4% paraformaldehyde in PBS. Non-migrated cells on the upper side of the filter were removed with a cotton swab. Cells on the underside of the filter were stained with 0.1% crystal violet for 20 minutes and then were eluted by 33% glacial acetic acid. OD values were read by Multiskan (Thermo) at 595nm. Relative cell migration was determined by the OD values of nifedipine groups normalized to the OD values of control groups.

### Calcium imaging

Calcium imaging was performed by using Fura-8 ™ (AAT Bioquest) according to previous description. Fluorescence images of the cells were recorded and analyzed with a video image analysis system.

### Immunoblotting

Cells were lysed ice-cold RIPA lysis buffer (Solarbio) including PMSF and proteinase inhibitor cocktail (Roche). Samples were centrifuged for 15min at 12000g at 4°C and the supernatants were harvested. About 100 ug of each protein sample were loaded and separated on 12% Bis-Tris Gel followed by transferring to 0.45 µm PVDF membrane. The membranes were then blocked with 5% non-fat dry milk, probed with appropriate primary antibodies, followed incubation by HRP-conjugated secondary antibodies at a dilution of 1: 3000. The following antibodies were used: rabbit anti-P(Ser473)-Akt (Cell Signaling, #9271), rabbit anti-total Akt (Cell Signaling, #9272), rabbit anti-P(Thr202/Tyr204)-Erk(Cell Signaling, #9101), mouse anti-total Erk (cell signaling, #4696), mouse anti-GAPDH (Beijing TDY Biotech LTD, #M010), mouse anti-β-actin (Beijing TDY Biotech LTD, #M009), HRP-conjugated secondary mouse(Beijing TDY Biotech LTD, #E009) and rabbit antibodies(Beijing TDY Biotech LTD, #E011).

### Detection of mRNAs and miRNAs

Total RNA from cells and tumor tissue samples were extracted using Trizol (Invitrogen). Chloroform was added to each sample and incubated for phase separation. Samples were centrifuged at 12,000 g for 15min at 4°C and the RNA (top aqueous phase) was isolated. RNA was precipitated by mixing with isopropanol gently and centrifuging at 12,000 g for 10min at 4°C. The RNA pellet was washed with 75% ethanol, dried, and dissolved in RNase-free water.

For mRNAs detection and expression, total RNA was reverse transcribed using random primers and cDNA synthesis Kit (TransGen Biotech). The resulting cDNAs were mixed with the SYBR PCR master mix (TransGen Biotech) and run on the Step-One Plus Applied Biosystems Real-time PCR machine. One cycle of denaturing step (30 sec at 94°C) was applied, followed by 40 cycles of amplification (5 sec at 94°C and 30sec at 60°C), with fluorescence measured during the extension. 18s was as the house keeping gene to normalize the gene expression. Primers used in this study are listed in the table S1.

For miRNAs detection, we employed All-in-One™ miRNA qRT-PCR Detection Kit (GeneCopoeia, #AOMD-Q020) according to the manufacturer’s instructions. The U6 small nuclear RNA was used as the control to determine relative miRNA expression.

The relative quantification value which reflected the fold changes of mRNAs/miRNAs expression in nifedipine group compared to control was calculated using the comparative CT (ΔΔC_T_) method and StepOne™ software v2.0.1 (Applied Biosystems). At least three independent experiments were performed to derive average relative quantification and SEM.

### Gene expression microarray analysis

The aminoallyl-RNA (aRNA) probes were amplified using the MessageAmp aRNA kit (Ambion, #1753) and labeled with the NHS-Cy5 (GE Amersham, USA, #PA25001). The Cy5-labeled RNA probes were further purified and quantified. The labeled probes were hybridized at 50°C for 16hours to the Human Whole Genome One Array TM Version 4.3(Phalanx Biotech Group, Taiwan) containing 30,968 well-characterized genes and then scanned with the Axon 4000B Scanner (Molecular Devices, USA) as well as analyzed with Genepix software (Molecular Devices USA).

### Plasmids, oligonucleotides and transfection

For 3′UTR reporter construction, the 3′UTR of BRI3 were PCR-amplified from human genomic DNA and cloned into the psiCHECK2 plasmid(Promega). The plasmid sequences were verified to be free of mutation by direct sequencing. BRI3 si-RNA, hsa-miR-524-5p mimics were chemically synthesized and purified by high-performance liquid chromatography (GenePharma, Shanghai, China). The sequences targeted to silence BRI3 mRNA (NM_015379) were: 5′-GAUUAAGCUUCAGGACUGUTT-3′(sense), 5′-ACAGUCCUGAAGCUUAAUCTT-3′ (antisense). Hsa-miRNA-524-5p sequence were: 5′-CUACAAAGGGAAGCACUUUCUC-3′ (sense), 5′-GAAAGUGCUUCCCUUUGUAGUU-3′ (antisense). Both mock and negative controls were used to assess the transfection. Mock group was only transfected with Lipofectamine 2000(Invitrogen). Negative control sequences: 5′-UUCUCCGAACGUGUCACGUTT-3′ (sense), 5′-ACGUGACACGUUCGGAGAATT-3′ (antisense).

### Statistical analysis

The Student’s t-test was used for the statistical analysis of all the independent experiments, with significance accepted at *P<0.05*. In the dependent experiments, such as the tumor volume analysis, Mixed Model in SAS software was used. A *p*-value of *<0.05* was considered statistically significant.

## Results

### Nifedipine stimulated the growth of breast cancer cell in the nude mice

**Figure 1:**
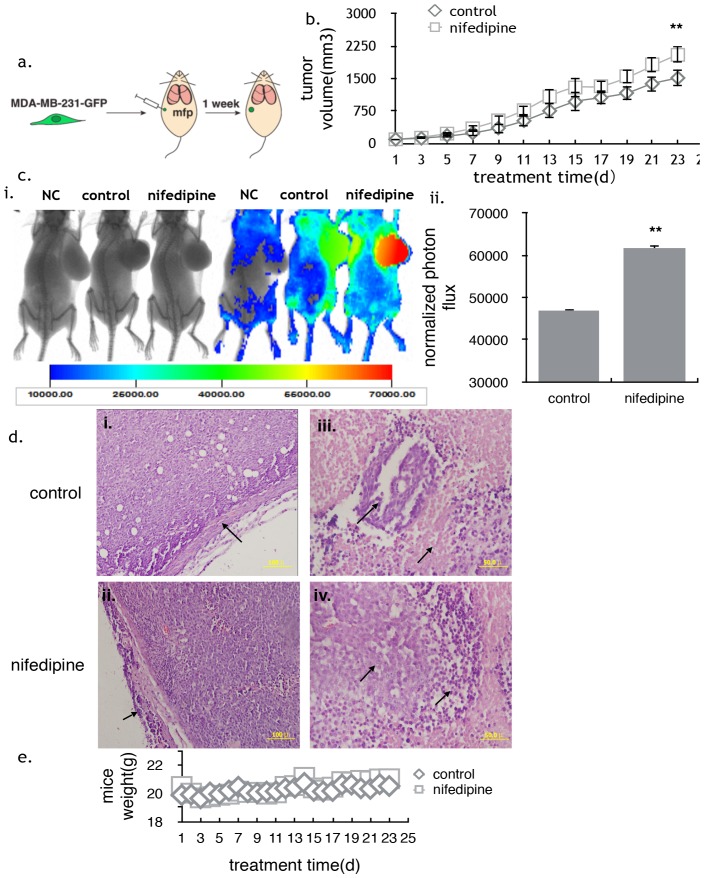
The effect of nifedipine on the nude mice *in* **vivo.** a) Nude mice were injected with 510^6^ MDA-MB-231-GFP cells into the second fat pads to imitate the orthotopic breast cancer inoculation. After one week injection, when the breast tumor size reached 100mm^3^, nude mice were then fed with nifedipine (4.8 mg/kg) or CMC-Na. b) The tumor volume in nude mice described in (a) was measured at the indicated time points and plotted as the mean volume ± SEM; n = 10; **P**<0.01, Mixed Model in SAS software. c) *In vivo* imaging of tumors treated with nifedipine or CMC-Na after three weeks(n = 10). i) Nifedipine groups had stronger fluorescence compared with control groups. ‘NC’ represented negative control and it was used to eliminate the background difference. ‘Control’ represented the mice treated with CMC-Na and ‘nifedipine’ represented the mice treated with nifedipine. ii) The normalized photon flux showed that the fluorescence increased about 30%. n = 10; P<0.01,1-way ANOVA. d) HE staining of xenograft in subcutaneous implanted tumor models after the treatment with nifedipine or CMC-Na. i) Control groups had complete envelope. Fewer cancer cells invaded the envelope. ii) Nifedipine groups had uncompleted envelope and tumor cells invaded in skeletal muscles. iii) Control groups had larger tumor cells necrosis and less tumor cells genesis. iv) Tumor cells necrosis and genesis happened at the same time in nifedipine groups (Fig. i and ii magnification ×20; Fig. iii and iv magnification ×40). e) Nifedipine has no effect the weight of nude mice. n = 10; P>0.05, Mixed Model in SAS software.

MDA-MB-231 breast cancer cells were transfected by PsinDest5.1-GFP to form MDA-MB-231-GFP cells with stable fluorescence. To induce breast cancer *invivo,* MDA-MB-231-GFP cells were injected into the second mamma fat pad (Fig. 1a). After inoculated for one week, breast tumors in nude mice developed to 100mm^3^. The tumor size was measured by vernier calipersize. Thereafter the nude mice were treated with nifedipine by intra-gastric administration every day. The control groups were fed with equivalent CMC-Na. As a result, after three weeks, the tumor size in the nifedipine-treated group was significantly bigger than that in the control groups (Fig. 1b), but there wasn’t significant alternation in the whole body weight among different groups (Fig. 1e). Vivo imaging also showed that the nifedipine-treated groups had stronger fluorescence than control after 3 weeks (Fig. 1c i&ii), indicating that tumors in the nifedipine-treated group were more active and invasive. Furthermore, a complete capsulewas predominantly observed in the subcutaneous implanted tumor nodes of the CMC-Na groups by HE staining of xenograft and no cancer cells observed outside the tumor node (Fig. 1d i). In addition, there were large tumor cells necrosis and but little tumor cells genesis (Fig. 1d iii). However, in nifedipine treated groups, tumor cells had invaded into skeletal muscles (Fig. 1d ii), and tumor cells genesis along with tumor cells necrosis was present (Fig. 1d iv). It was concluded that tumor grew bigger and the fluorescence became stronger in the nifedipine-treated group after two and four weeks (Fig. S1).

### Nifedipine promoted proliferation and migration of breast cancer cells invitro

**Figure 2:**
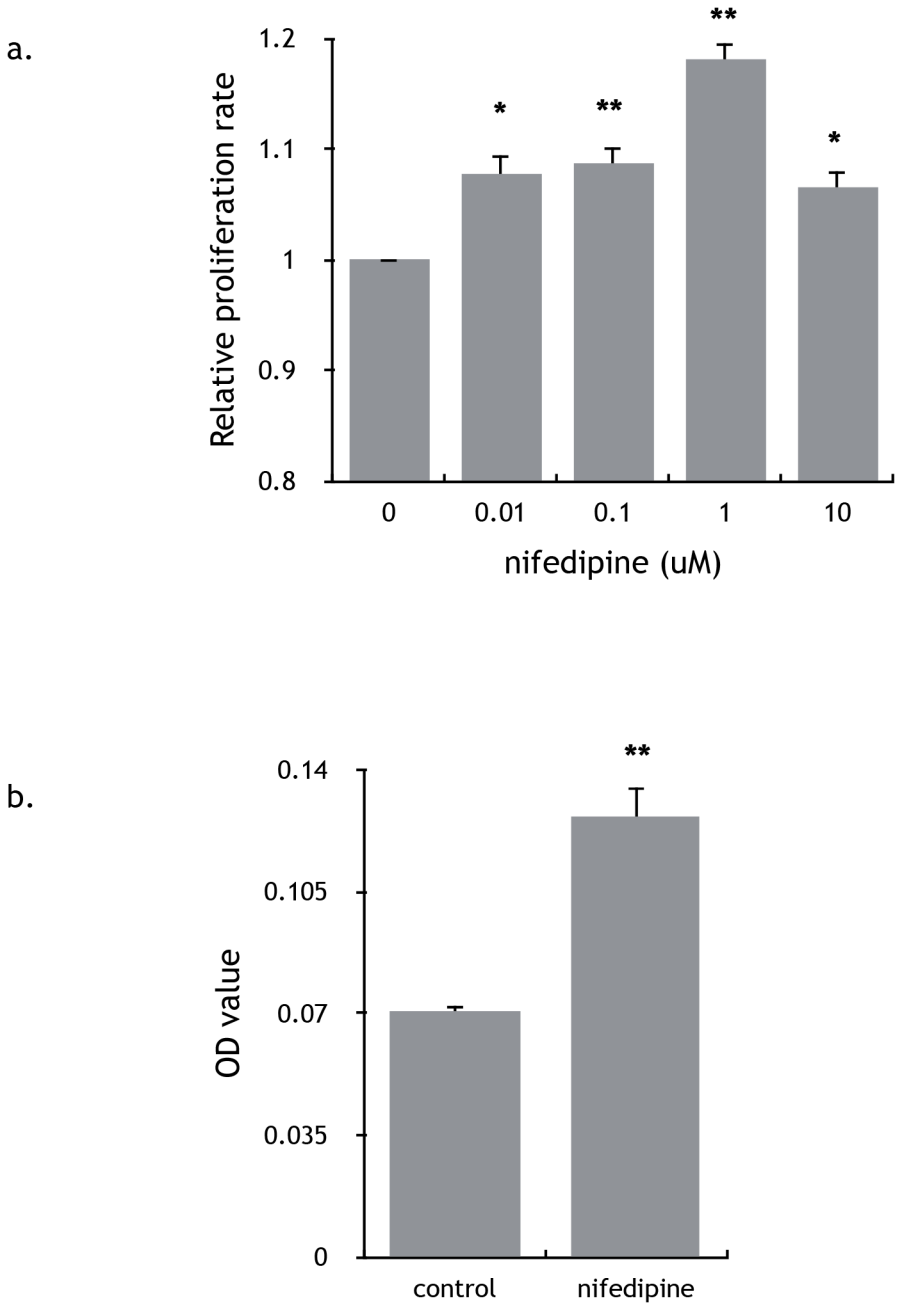
Nifedipine stimulated the survival and migration of breast cancer cells *invitro*. a) Different concentrations of nifedipine could stimulate the proliferation of MDA-MB-231 cells. Results represented mean ± SEM of 3 independent experiments. P<0.01, 1-way ANOVA. b) Nifedipine (1 µM) promoted the migration of MDA-MB-231 cells. Results represented the mean ± SEM in 3 independent experiments. P = 0.003, 1-way ANOVA.

To further investigate the effect of nifedipine on breast cancer cells, MDA-MB-231 breast cancer cells were treated with nifedipine at different concentrations *invitro*. As measured by MTT assay, nifedipine significantly promoted the proliferation of MDA-MB-231 cells and it induced the strongest proliferation at the concentration of 1 µM (Fig. 2a). MDA-MB-231 cells also showed significant increased migration in response to 1 uM nifedipine. The increased migration rate is nearly two fold of the control (Fig. 2b).

In consistent with those findings, nifedipine exhibited the same effects on another breast cancer cell line, MCF-7. Nifedipine also significantly promoted the proliferation and migration of MCF-7 cells at the concentration of 10 µM (Fig. S2).

### Verapamil, another CCBs, had no effect on the tumor growth *invivo*

**Figure 3:**
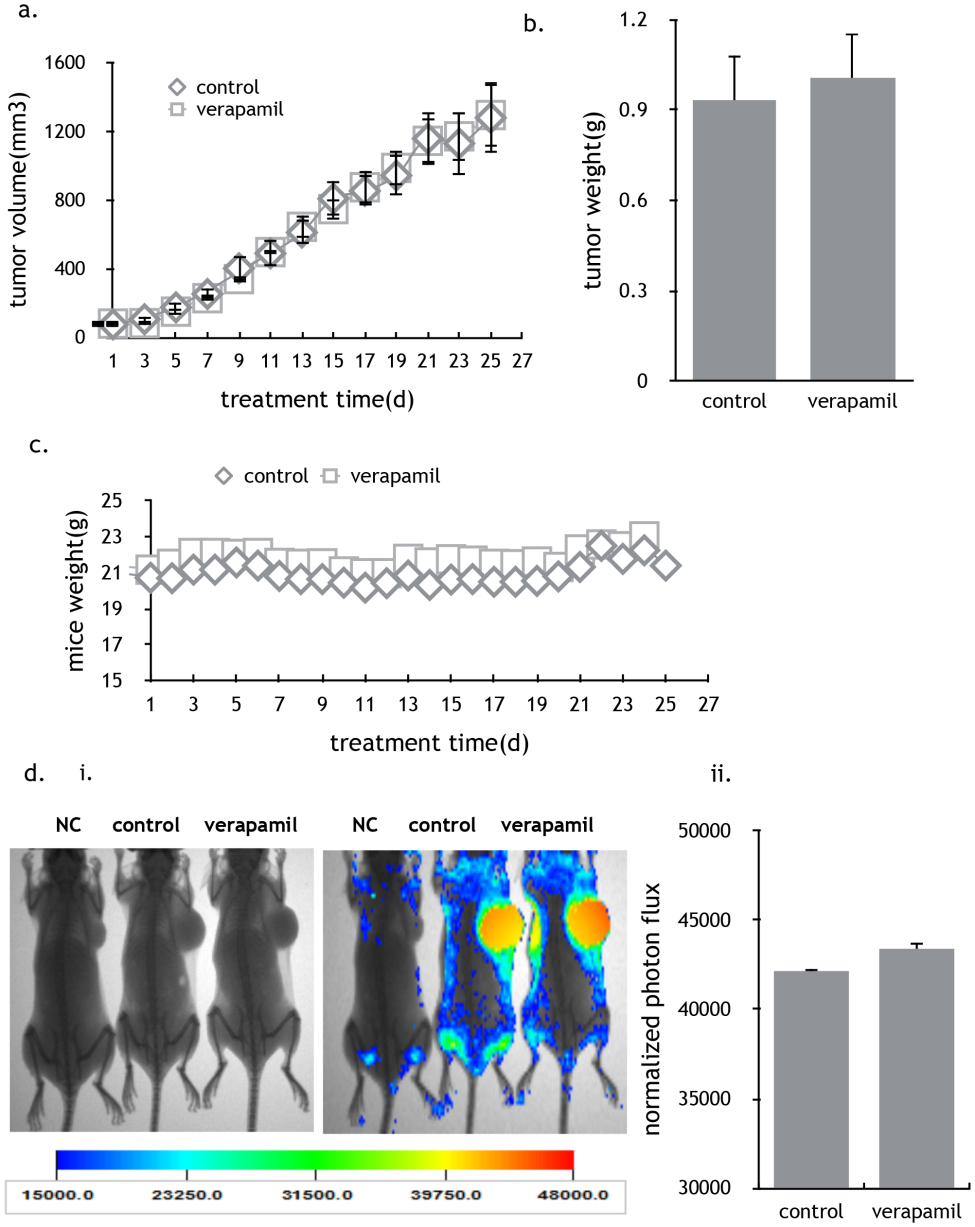
Verapamil, another CCB, had no effect the tumor growth invivo. a) Verapamil had no obvious effect on tumor size in nude mice injected with 5⋅10^6^ MDA-MB-231-GFP cells. Results represented the mean ± SEM. n = 10; P>0.05, Mixed Model in SAS software. b) Effect of different periods with verapamil on the tumor weight. No obvious difference was observed. Results were represented the mean ± SEM. n = 10; P>0.05, 1-way ANOVA. c) Verapamil had no effect on the weight of nude mice compared with the control. n = 10; P>0.05, Mixed Model in SAS software. d) *In vivo* imaging of the tumors with the treatment of H_2_O and verapamil. i) Verapamil showed no effect on the tumor growth. ii) The normalized photon flux also showed no difference between verapamil treated mice and control.

Nifedipine is one of the CCBs that are used to treat hypertension. To test whether the stimulation of breast tumor is nifedipine specific, another CCBs, verapamil, was used to treat nude mice infected with MDA-MB-231-GFP cells. However no significant change in tumor size (Fig. 3a), tumor weight (Fig. 3b) and mice weight (Fig. 3c) was observed. In addition, the fluorescence intensity exhibited no difference between groups treated with nifedipine and CMC-Na (Fig. 3d). Thus the specific effects of nifedipine on MDA-MB-231 cells were not a common character of L-type calcium channel blockers.

### Effect of nifedipine not due to intracellular Ca^2+^ in MDA-MB-231 cells

**Figure 4:**
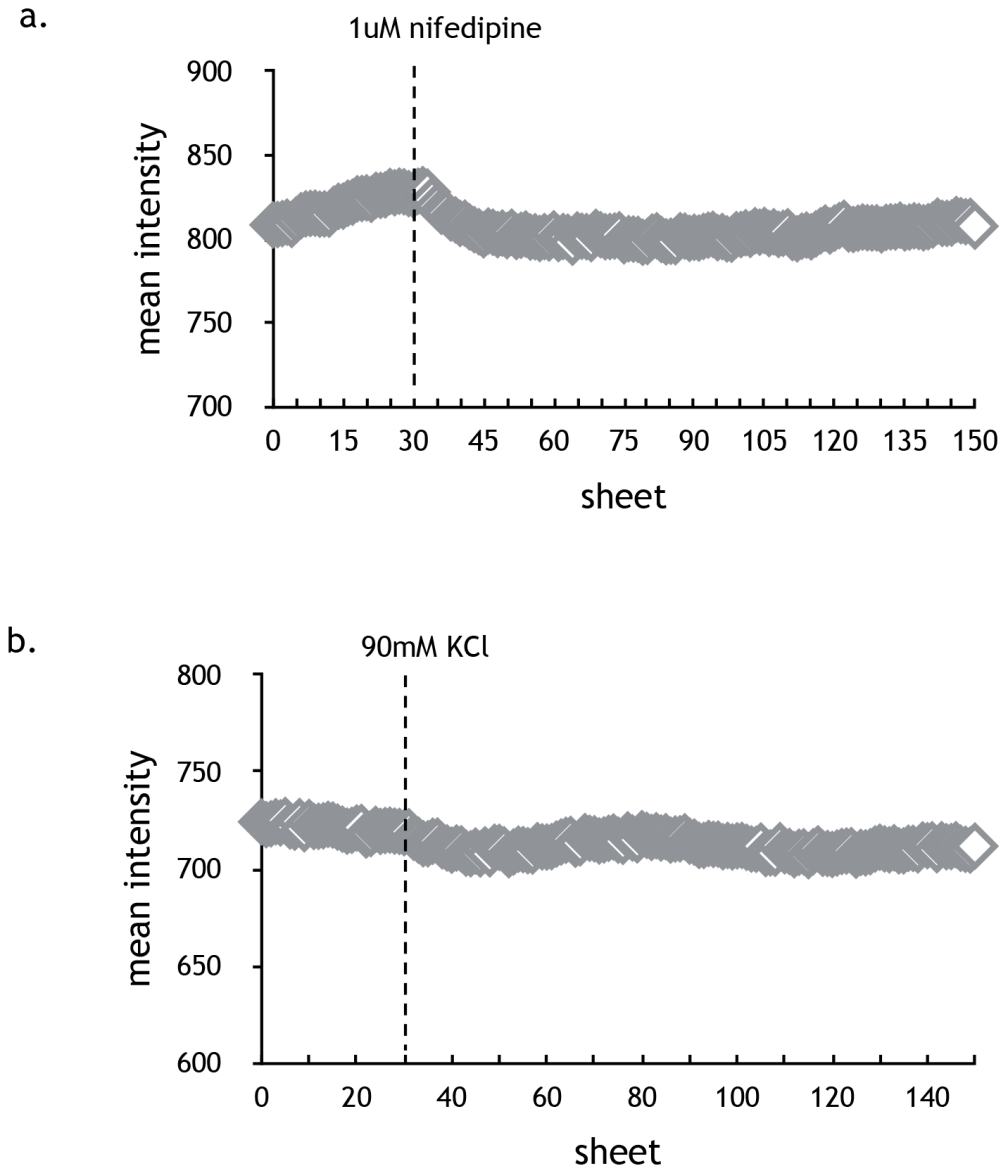
Effect of nifedipine on intracellular calcium concentration in MDA-MB-231 cells. (a) MDA-MB-231 breast cancer cells were loaded with Furo-8 for 30 minutes, as described in “Materials and Methods”. Base-line [Ca^2+^]_i_ was recorded, followed by sequential additions of 1 uM nifedipine. No obvious changes were observed until the 150th picture was taken (n = 300). (b) No significant increase in [Ca^2+^]_i_ was observed by increasing the bath [K^+^] from 2.5 mM to 90mM until the 150th picture was taken (n = 300).

To test whether the effect of nifedipine on proliferation and migration of breast cancer cells is related to the concentration of the intracellular free [Ca^2+^], the MDA-MB-231 cells were preincubated in the presence of 1 µM nifedipine in L-15 containing 10% FBS for 1h at 37°C and then loaded with Furo-8 as described in “Materials and Methods”. No significant increase in [Ca^2+^]_i_ was observed by either nifedipine alone (Fig. 4a) or increasing the bath [K^+^] from 2.5 mM to 90mM (Fig. 4b), indicating that MDA-MB-231 cells didn’t express functional L-type calcium channel. It suggested that effect of nifedipine on the proliferation and migration of MDA-MB-231 cells was not related with calcium channel and cellular [Ca^2+^].

### Nifedipine activated signaling pathways to promote cell growth in breast cancer cells

**Figure 5:**
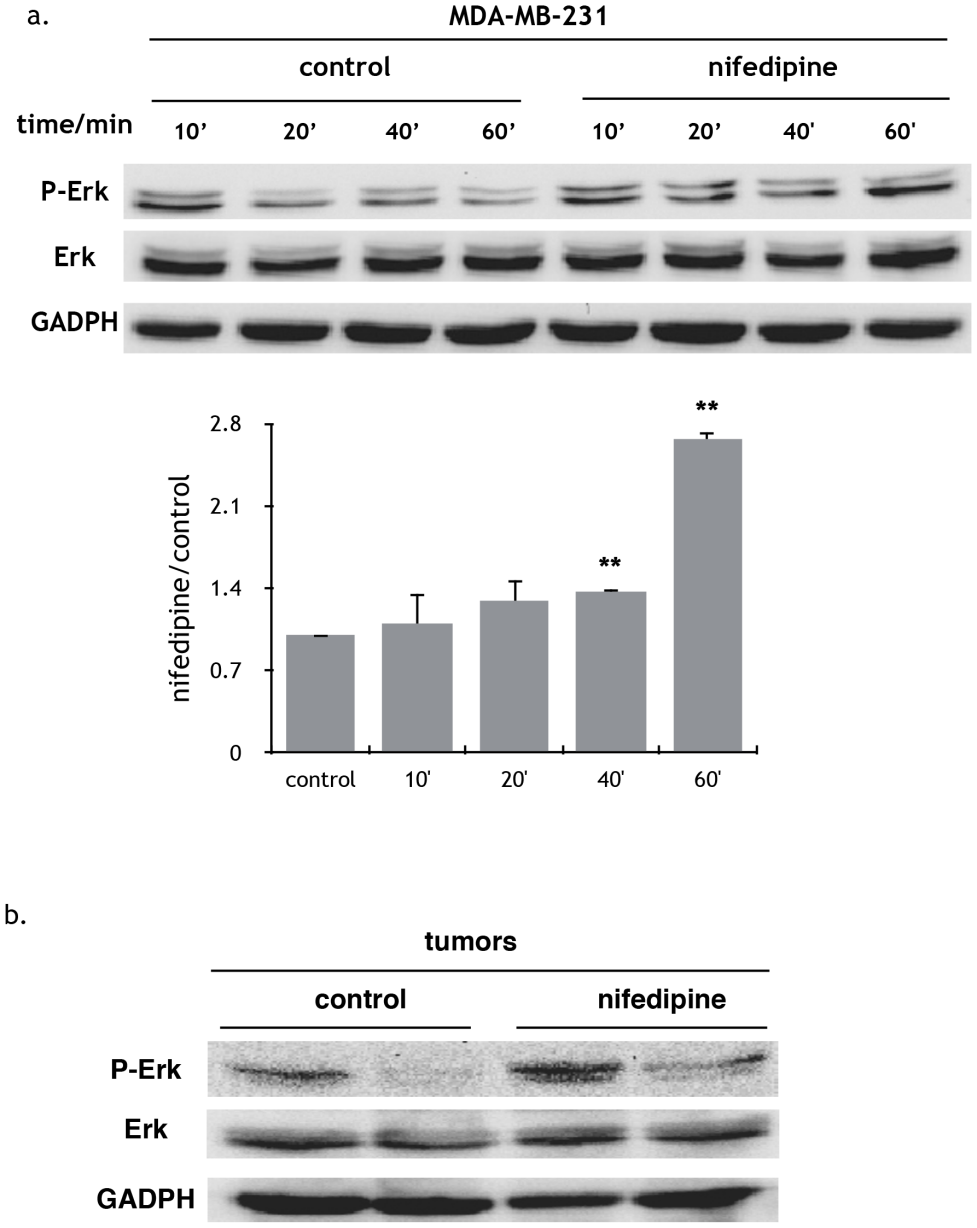
ERK activation in Nifedipine treated breast cancer cells. Phosphorylated Erk (P-Erk) immunoblotting in MDA-MB-231 cells treated with or without nifedipine at the indicated times. The level of phosphorylation was more intensive after treated with nifedipine for 40 minutes. Membranes were reprobed for GADPH for loading control. Gray value was analyzed by the Image J software. Results were represented the mean±SEM. n = 3; 1-way ANOVA. b) Phosphorylated Erk (P-Erk) immunoblotting in tumor tissues from nude mice treated with CMC-Na or nifedipine for 4 weeks. Membranes were reprobed for GADPH for loading control. The results were consistent with that in the MDA-MB-231 cells.

To elucidate the signaling pathways underlying the effects of nifedipine on breast cancer cells, key factors involved in growth control of cancer cells were examined. Strikingly, increase of phosphorylated Erk was found both in MDA-MB-231 cells exposure to nifedipine (Fig. 5a) and in MDA-MB-231 tumor tissue from nude mice treated with nifedipine (Fig. 5b).

### Selection and validation of BRI3 as the candidate biomarkers related to nifedipine

**Figure 6:**
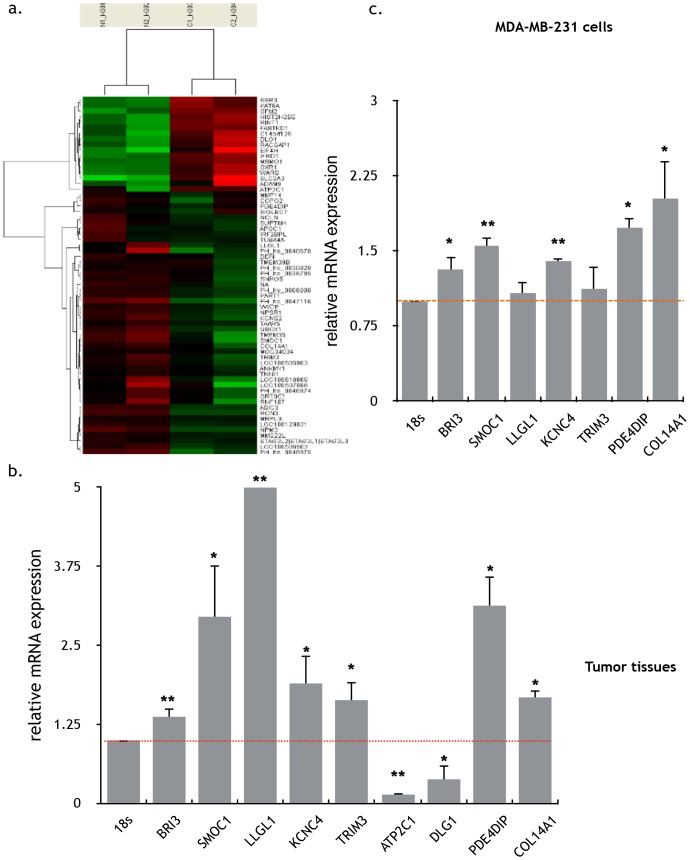
Selection and validation of the candidate biomarkers related to nifedipine. a) Comparison of gene expression profiles from nude mice tumors with the treatment of nifedipine or CMC-Na. Hierarchical clustering of nude mice tumors with different treatments. A dendrogram of the tumors was shown at the top. The changed genes were also clustered and gene names were on the right. Taken together, there were 51 up-regulated and 18 down-regulated genes found. Among them, there were 35 genes related to cell adhesion and migration. b) Transcript levels of differential genes in nude mice tumors treated with nifedipine or CMC-Na. Nine genes (BRI3, SMOC1, LLGL1, KCNC4, TRIM3, ATP2C1, DLG1, PDE4DIP and COL14A1) were found to have a consistent gain or loss in at least 10 tumor samples. The transcript levels were normalized to the expression of internal 18s rDNA. Data were presented as the mean ± SEM of three independent experiments. Specific comparison between nifedipine groups and control groups, P<0.01, 1-way ANOVA. c) Transcript levels of differential genes in MDA-MB-231 cells treated with nifedipine for different periods by real-time PCR. KCNC4, BRI3, SMOC1, PDE4DIP and COL14A1 genes were found to have a consistent gain or loss in the MDA-MB-231 cells treated with nifedipine. Results were presented as the mean ± SEM of three independent experiments. Specific comparison between nifedipine groups and control groups, **,P<0.01; *, P<0.05,1-way ANOVA.

**Figure 7:**
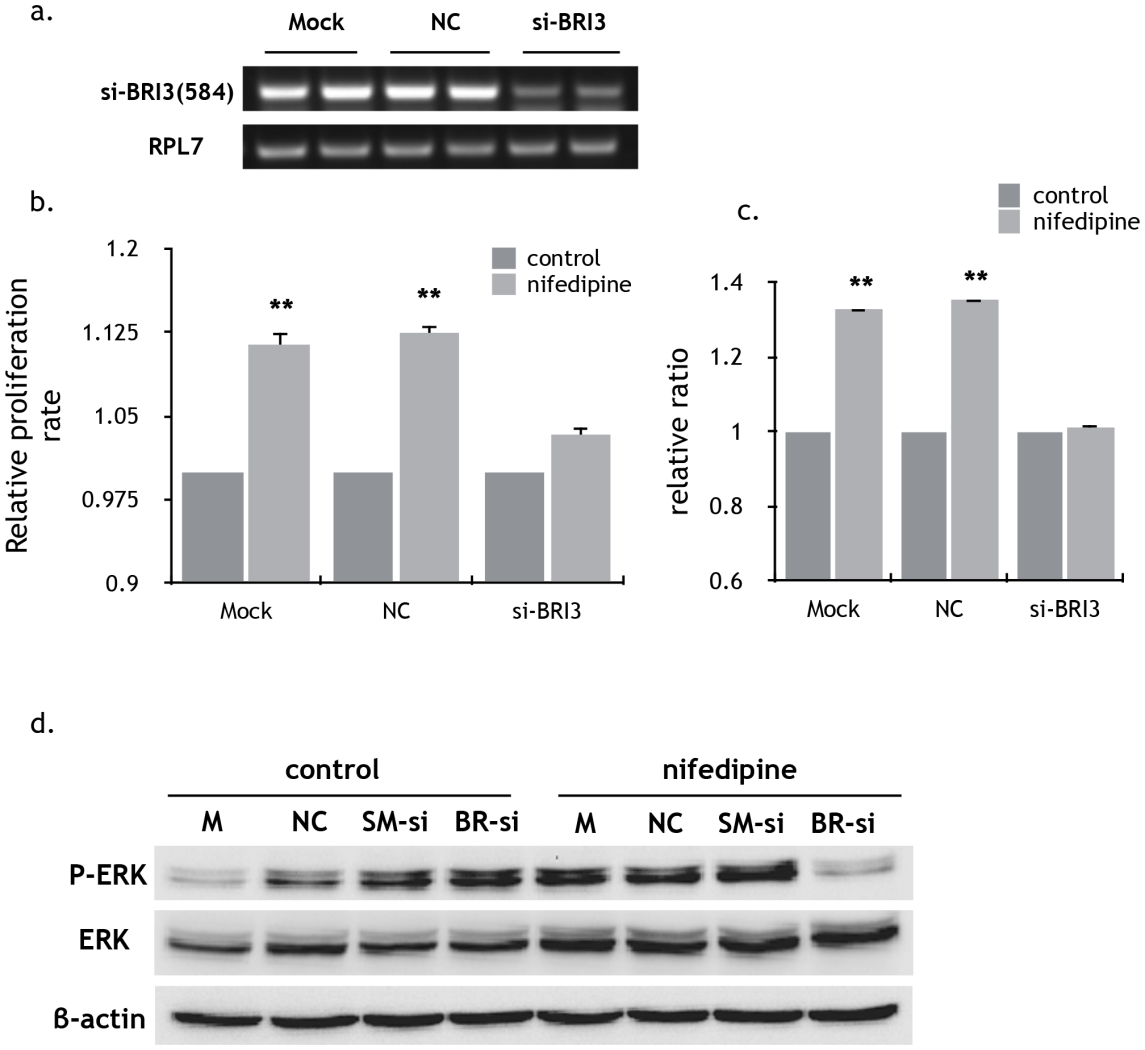
Inhibition of proliferation, migration and P-Erk of MDA-MB-231 by silencing BRI3 gene expression. a) Effective silencing of BRI3 mRNA in MDA-MB-231 cells after siRNA treatment. BRI3-homo-584 siRNA markedly inhibited BRI3 mRNA expression at 100 nM after transfection for 24h. b) siRNA directed against BRI3 suppressed the facilitation of nifedipine in the proliferation of MDA-MB-231 cells. Data were presented as the mean±SEM of three independent experiments. Specific comparison between nifedipine groups and control groups, P<0.01, 1-way ANOVA. c) MDA-MB-231 cells transfected with mock, si-BRI3 and negative control were plated in the upper chamber of transwell. Data were presented as the mean ± SEM of three independent experiments. Specific comparison between nifedipine groups and control groups, P<0.01, 1-way ANOVA. d) Increased protein expression of P-Erk in MDA-MB-231 cell lines treated with nifedipine was inhibited 72h after transfection of BRI3-siRNA. M means mock, NC means negative control, SM-si means si-SMOC1 gene, BR-si means si-BRI3 gene.

To find out the candidate targets of nifedipine regarding to its function on breast cancer cells, tumor tissues from nude mice were used for gene expression analysis by microarray (Fig. 6a). In tumor tissue gene expression of 51 genes was found significantly up-regulated (Table. S2) and down-regulated in 18 genes (Table. S3). Among them, there were 35 genes whose functions are related to cell adhesion and migration (Table. S4) analyzed using DAVID (http://david.abcc.ncifcrf.gov/home.jsp) for gene ontology annotation. Using real-time PCR, expression of 9 interested genes was validated in the tumor tissues (Fig. 6b). Among them, 5 genes also had the similar variation trend in MDA-MB-231 cells treated with nifedipine (Fig. 6c). They included BRI3, SMOC1, KCNC4, PDE4DIP and COL14A1. To determine the role of BRI3, SMOC1, KCNC4, PDE4DIP and COL14A1 in tumor proliferation and migration, siRNAs were constructed to silencing the expression of these genes in breast cancer cells. Silencing of BRI3 expression (Fig. 7a) could markedly decrease the proliferation and migration (Fig. 7b, c) and suppress Erk phosphorylation in MDA-MB-231 cell lines treated with nifedipine (Fig. 7d). These results indicate that BRI3 is an important target related to the function of nifedipine in the proliferation and migration of MDA-MB-231 breast cancer cells. We also confirmed that nifedipine could increase the expression of ANGPTL7 gene in the tumor [20] and MDA-MB-231 cells when treated with nifedipine for 48h compared with control (Fig. S3).

### miRNA-524-5p acts as the upstream of BRI3 and indirectly regulated BRI3

**Figure 8:**
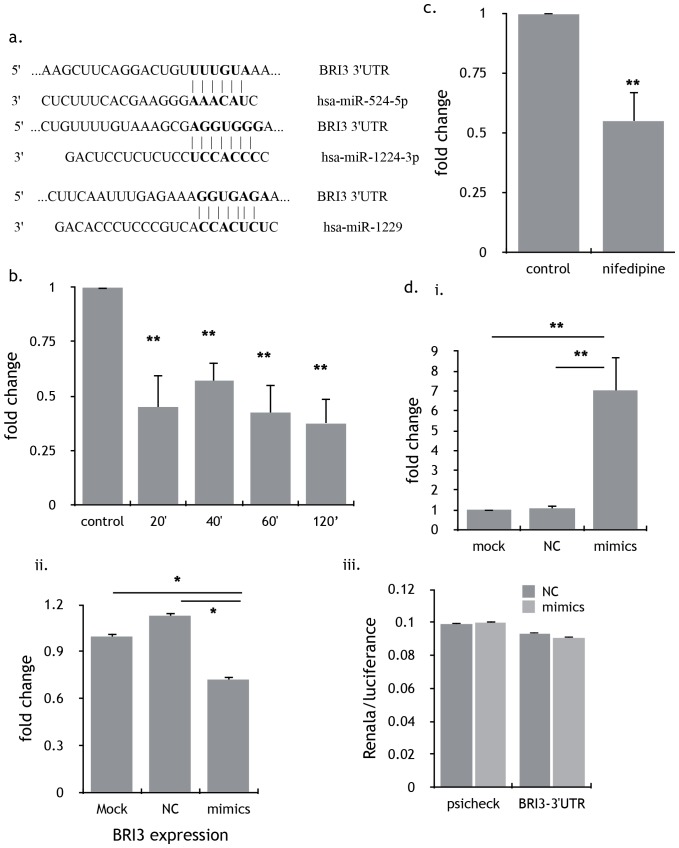
miRNA-524-5p was target of nifedipine and regulated the expression of BRI3. a) Predicted duplex formation between human BRI3 3′-UTR (top) and three miRNA (bottom) including has-miRNA-524-5p, hsa-miR-1224-3p, hsa-miR-1229 by using TargetScan software. b) The expression of miRNA-524-5p decreased in the MDA-MB-231 cells treated with nifedipine for different periods. Results were presented as the mean±SEM of three independent experiments. P<0.01, 1-way ANOVA. c) The expression of miRNA-524-5p decreased in the tumor tissues from nude mice fed with nifedipine compared to CMC-Na groups. Results were presented as the mean±SEM of three independent experiments. P<0.05, 1-way ANOVA. d) Overexpression of miRNA-524-5p could indirectly decrease the expression of BRI3 gene. i) MDA-MB-231 cells were transfected with miRNA-524-5p mimics and negative control (both 100 nM) for 24h. To test the expression of miRNA-524-5p by qPCR and the expression of miRNA-524-5p increased about 8 times. Results were presented as the mean±SEM of three independent experiments. P<0.01, 1-way ANOVA. ii) The expression of BRI3 gene decreased obviously compared with negative control when miRNA-524-5p overexpressed. Results were presented as the mean±SEM of three independent experiments. P<0.05, 1-way ANOVA. iii) Relative luciferase activity (Renilla luciferase/Firefly luciferase) levels were measured after 24h from co-transfection of MDA-MB-231 cells with indicated BRI3-3′UTR-psiCHECK2 plasmid with miRNA-524-5p mimics or NC. The relative luciferase activity has no change. Results were presented as the mean±SEM of three independent experiments. P>0.05,1-way ANOVA.

To seek the target miRNA of BRI3 we performed a TargetScan analysis and found miR-524-5p, miR-1224-3p and miR-1229 as candidate target miRNA of BRI3 based on a TargetScan analysis (Fig. 8a). However only miR524-5-p was eventually confirmed as a regulator of BRI3 as only the expression of miRNA-524-5p decreased when MDA-MB-231 cells was treated with nifedipine for different periods (Fig. 8b); but not the expression of miR-1224-3p and miR-1229 (data not shown). Expression of miRNA-524-5p also decreased for nearly 2 folds in tumor tissues from the nude mice (Fig. 8c). Furthermore overexpression of miRNA-524-5p (Fig. 8d i) obviously decreased the expression of BRI3 gene (Fig. 8d ii), which indicates miRNA-524-5p acting as upstream of BRI3. We inserted the 3′UTR of BRI3 gene to psiCHECK2 plasmid and tested the Renilla luciferase activity. As a result, the Renilla/Firefly activity was no change by adding miRNA-524-5p mimics (Fig. 8d iii). Overexpression of mi-RNA-524-5p also decreased the expression of PDE4DIP, SMOC1 and COL14A1 (Fig. S4).

## Discussion

In this study, we found that nifedipine significantly stimulated breast cancer growth in the nude mice without any effects on the mice weight. *In vivo* imaging of tumor tissue in nude mice showed that nifedipine treated mice had stronger and wider range of fluorescence, suggesting tumors were more active and easier to migrate. The pathological section confirmed the hypothesis. CMC-Na groups had complete envelopes and large tumor cells necrosis in the middle of tumors; whereas cancer cells invaded skeletal muscles in the nifedipine treatment groups. Additionally, nifedipine promoted the proliferation and migration of both MDA-MB-231 and MCF-7 cells by *in-vitro* and *in-vivo* assay. However, verapamil, another calcium channel blocker, didn’t have the similar effects in nude mice.

Previous studies have resulted in a controversial conclusion on whether CCBs promote cancer cells. Our results confirmed that nifedipine can potentiate the breast cancers. With respect to the possible mechanism, [Ca^2+^]_i_ modulation was excluded in the first instance. MDA-MB-231 cells don’t express the CACNA1C and CACNA1D subtypes (data not shown), which is consistent with the previous report [17]. Moreover, that 1 µM nifedipine failed to alter [Ca^2+^]_i_, ruled out the connection between calcium and the promotion effect of nifedipine. The lack of expression of voltage gated calcium channels (VGCCs) in breast cancer cells likely counts upon the rationale that blockers of VGCCs should have no effect on breast cancers. Inconsistent to most previous studies, nifedipine exerts the distinct effect from verapamil, suggesting the specificity of nifedipine on its promotion outcome instead of the character of general blockers of CCBs. Verapamil was even reported to inhibit the growth of cancer cell [17]. Different compound structures and binding motifs may explain the different effects of CCBs on cancer cells.

Nifedipine activated the phosphorylation of Erk in MDA-MB-231 cells both *invivo* and *invitro*, which suggests it functions through Erk signaling pathway. Referring to the target genes in the upstream of Erk, micro-array and Q-PCR analysis from both *in* *vivo* tumor tissues and *invitro* cancer cells consistently reflected 9 interested genes. Among them, SMOC1, KCNC4, PDE4DIP and COL14A1 have been reported to associate with a variety of tumors. SMOC1 is SPARC (secreted protein, acidic and rich in cysteine) related modular calcium binding 1[21], [22] and has been found a correlation with malignant progression, such as brain tumor, colorectal cancer and breast cancer [23]. KCNC4 (potassium voltage-gated channel, Shaw-related subfamily) is a potassium voltage-gated channel and the expression increase of Kv3.4 can promote the proliferation of OSCC [24]. PDE4DIP (also known as myomegalin, MMGL) is a tumor marker for diagnose and establish a prognosis in patients with esophageal squamous cell carcinoma [25]. COL14A1(collagenfamily) may be involved in the basement membrane regulation, providing specific molecular bridges between fibrils and other matrix components [26]. However, our work revealed that BRI3 is closely related with this nifedipine effect on breast cancers. BRI3 participates in tumor necrosis factor- induced cell death [26], [27]. Silencing of BRI3 expression clearly suppressed the phosphorylation of Erk, and consequently the proliferation and migration of MDA-MB-231 cells, suggesting that the BRI3-Erk signaling pathway is involved in regulation of this nifedipine effect on breast cancers.

As another mechanism underlining nifedipine promoting the breast cancer cell migration, up-regulation of ANGPTL7 was found in response to the nifedipine treatment. ANGPTL7 is a member of angiopoietin family as vascular regulators [20] which functions as a general endothelial cell survival factor [28] and modulates endothelial cell adhesion [29]. Previous work has shown that ANGTL can cause the gap between endothelial cells resulting in the metastasis of breast cancers [20].

MicroRNAs (miRNAs) are small, single-stranded, non-coding RNAs that regulate gene expression[30]. Their dysregulation therefore contributes to cancer cells’ proliferation and migration. For example, miRNA-10b initiated breast tumor invasion and metastasis [31]. miRNA-135a promoted breast cancer cell migration and invasion by targeting HOXA10 [32]. Restoration of miRNA-145 suppresses prostate cancer cell proliferation, migration and invasion by targeting FSCN1 [33]. In our study, expression of miRNA-524-5p decreased after cells were treated with nifedipine and miRNA-524-5p regulated the expression of BRI3 gene. Thus the effects of nifedipine on breast cancer is carried out by miRNA-524-5p-BRI3–Erk signaling pathway.

The women occupy about 1/3 in all the hypertension patients. Some of them are suffering or are genetically easier to develop breast cancers. Thus it is urged that prescription of nifedipine to women should be seriously caution. Nifedipine is dangerous for patients with breast cancers and it may worsen the situation. As a result, doctors should be aware of the fact that nifedipine promotes the breast cancers and avoid nifedipine for the women especially who suffer both breast cancer and hypertension.

## Supporting Information

Figure S1 **The effect of nifedipine on nude mice with different treatment time.** a) Nifedipine could increase the breast tumor volume and the facilitation was more stronger at the 4 weeks than at 2 weeks. n = 10 in each group; Results represented the mean±SEM (standard error). P<0.01, Mixed model in SAS software. b) Nifedipine could increase the breast tumor weight both in two weeks and four weeks. n = 10 in each group; Results represented the mean±SEM (standard error). P<0.01, Mixed model in SAS software. c) In vivo imaging of the tumors treated with nifedipine for different times. n = 10 in each group. i) As the time went on, nifedipine groups had more stronger fluorescence than control groups both in 2 weeks and 4 weeks. ii) The normalized photon flux showed the results in figure. d) Nifedipine couldn’t effect the weight of nude mice compared with the control groups at the 2 weeks or 4 weeks. n = 10 in each group; P>0.05, Mixed model in SAS software. e) HE staining of xenograft in subcutaneous implanted tumor models after the treatment with nifedipine or CMC-Na. Fig. i and ii were from the tumors treated with CMC-Na or nifedipine for two weeks. i) The envelope was complete in control groups and ii) it was becoming thin in nifedipine groups. Fig. iii–vi were from tumors treated for 4 weeks. iii) The envelope was thinner and v) more cells necrosis in control groups. iv) Meanwhile, tumor cells invaded seriously in envelope and vi) less cancer cells necrosis in nifedipine groups. (Fig. i,ii,v and vi magnification ×10; Fig. iii and iv magnification ×20).
(TIF)

Figure S2 **The effect of nifedipine on another breast cancer cell, MCF-7.** a) Nifedipine could promote the proliferation of MCF-7 cells at the concentration of 10 uM and the proliferation rate was about 10%. n = 32 per group; Results represented mean±SEM of 3 independent experiments. P<0.01,1-way ANOVA. b) Nifedipine(10 uM) promoted the migration of MCF-7 cells. Results represented the mean±SEM in 3 independent experiments. P = 0.0008, 1-way ANOVA.
(TIF)

Figure S3 **The effect of nifedipine on the expression of ANGPTL7 gene.** (A) The expression of ANGPTL7 increased nearly 3 times in the tumor tissues from nude mice treated with nifedipine. Results were presented as the mean±SEM of three independent experiments. *, P<0.05,1-way ANOVA. (B) The expression of ANGPTL7 increased in the MDA-MB-231 cells treated with nifedipine for 48h. Results were presented as the mean±SEM of three independent experiments. **, P<0.01,1-way ANOVA.
(TIF)

Figure S4 **miRNA-524-5p influenced the expression of other genes.** (A) Overexpression of miRNA-524-5p by adding the miRNA mimics also could decrease the expression of other genes, such as PDE4DIP, SMOC1 and COL14A1. Results were presented as the mean±SEM of three independent experiments. **, P<0.01,1-way ANOVA.
(TIF)

Table S1 **q-PCR primer list used in the study.**
(PDF)

Table S2 **Up-regulated genes from nude mice tumors with the treatment of nifedipine compared with CMC-Na.**
(PDF)

Table S3 **Down-regulated genes from nude mice tumors with the treatment of nifedipine compared with CMC-Na.**
(PDF)

Table S4 **35 genes related to cell adhension and migration of all the 69 changed genes.**
(PDF)
