# Supplementary figures and images for: Nifedipine Promotes the Proliferation and Migration of Breast Cancer Cells
Source: PLoS One. 2014 Dec 1;9(12):e113649. doi: 10.1371/journal.pone.0113649 (PMC4249963; doi:10.1371/journal.pone.0113649)

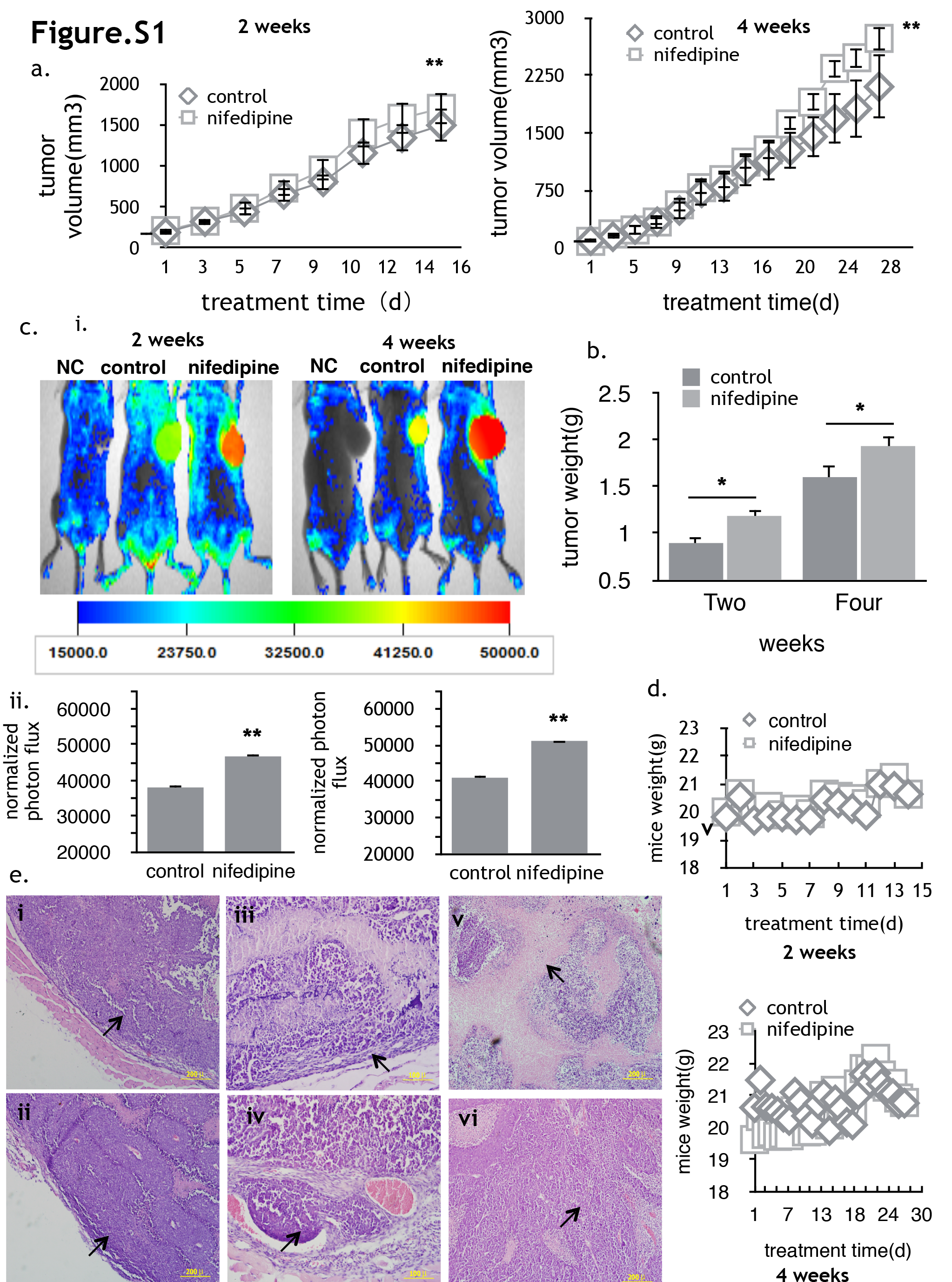

Supplement: Figure S1 — The effect of nifedipine on nude mice with different treatment time. a) Nifedipine could increase the breast tumor volume and the facilitation was more stronger at the 4 weeks than at 2 weeks. n = 10 in each group; Results represented the mean±SEM (standard error). P<0.01, Mixed model in SAS software. b) Nifedipine could increase the breast tumor weight both in two weeks and four weeks. n = 10 in each group; Results represented the mean±SEM (standard error). P<0.01, Mixed model in SAS software. c) In vivo imaging of the tumors treated with nifedipine for different times. n = 10 in each group. i) As the time went on, nifedipine groups had more stronger fluorescence than control groups both in 2 weeks and 4 weeks. ii) The normalized photon flux showed the results in figure. d) Nifedipine couldn’t effect the weight of nude mice compared with the control groups at the 2 weeks or 4 weeks. n = 10 in each group; P>0.05, Mixed model in SAS software. e) HE staining of xenograft in subcutaneous implanted tumor models after the treatment with nifedipine or CMC-Na. Fig. i and ii were from the tumors treated with CMC-Na or nifedipine for two weeks. i) The envelope was complete in control groups and ii) it was becoming thin in nifedipine groups. Fig. iii–vi were from tumors treated for 4 weeks. iii) The envelope was thinner and v) more cells necrosis in control groups. iv) Meanwhile, tumor cells invaded seriously in envelope and vi) less cancer cells necrosis in nifedipine groups. (Fig. i,ii,v and vi magnification ×10; Fig. iii and iv magnification ×20). (TIF) [file pone.0113649.s001.tif]

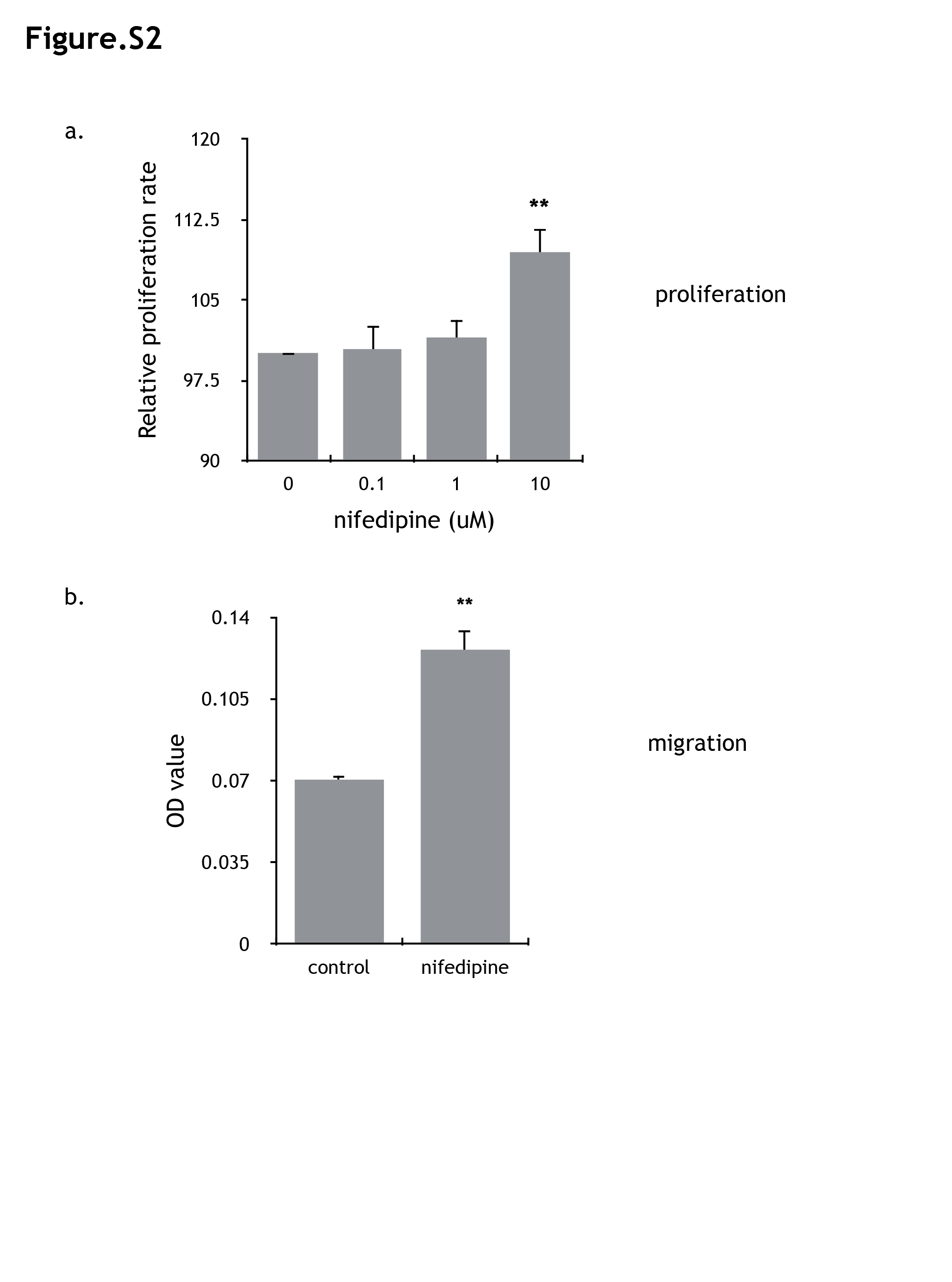

Supplement: Figure S2 — The effect of nifedipine on another breast cancer cell, MCF-7. a) Nifedipine could promote the proliferation of MCF-7 cells at the concentration of 10 uM and the proliferation rate was about 10%. n = 32 per group; Results represented mean±SEM of 3 independent experiments. P<0.01,1-way ANOVA. b) Nifedipine(10 uM) promoted the migration of MCF-7 cells. Results represented the mean±SEM in 3 independent experiments. P = 0.0008, 1-way ANOVA. (TIF) [file pone.0113649.s002.tif]

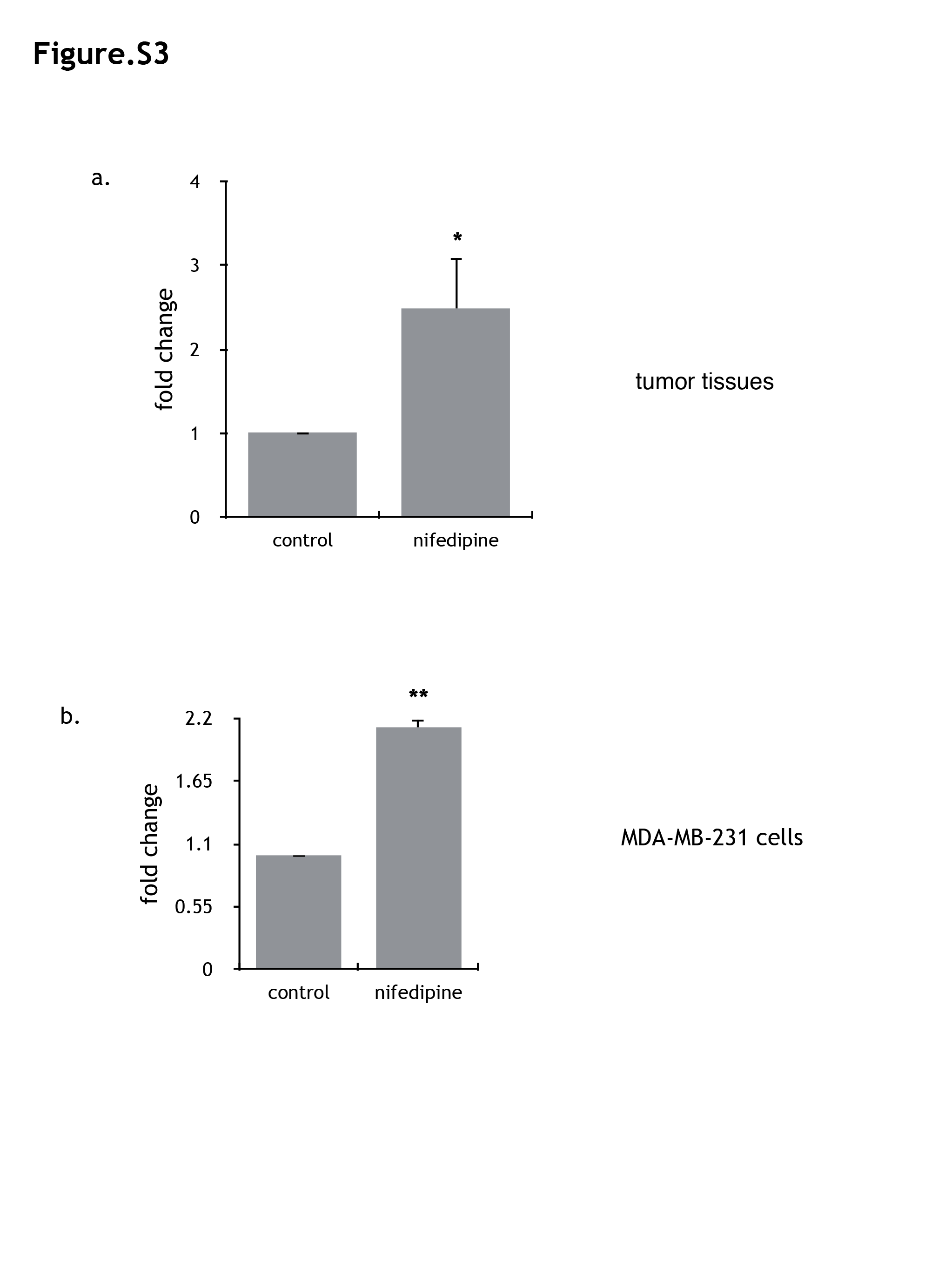

Supplement: Figure S3 — The effect of nifedipine on the expression of ANGPTL7 gene. (A) The expression of ANGPTL7 increased nearly 3 times in the tumor tissues from nude mice treated with nifedipine. Results were presented as the mean±SEM of three independent experiments. *, P<0.05,1-way ANOVA. (B) The expression of ANGPTL7 increased in the MDA-MB-231 cells treated with nifedipine for 48h. Results were presented as the mean±SEM of three independent experiments. **, P<0.01,1-way ANOVA. (TIF) [file pone.0113649.s003.tif]

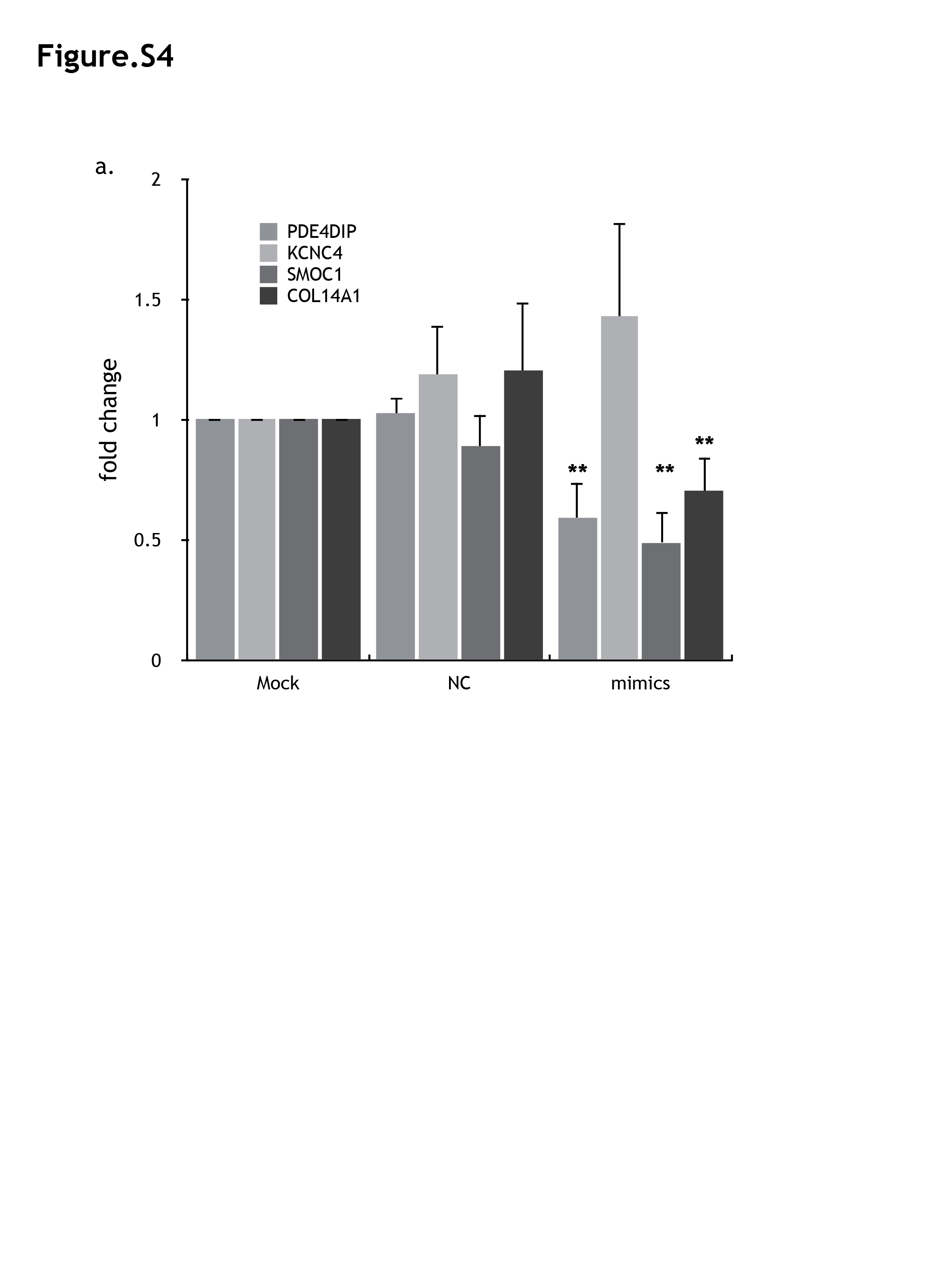

Supplement: Figure S4 — miRNA-524-5p influenced the expression of other genes. (A) Overexpression of miRNA-524-5p by adding the miRNA mimics also could decrease the expression of other genes, such as PDE4DIP, SMOC1 and COL14A1. Results were presented as the mean±SEM of three independent experiments. **, P<0.01,1-way ANOVA. (TIF) [file pone.0113649.s004.tif]
