# Supplementary material for: Nifedipine Promotes the Proliferation and Migration of Breast Cancer Cells
Source: PLoS One. 2014 Dec 1;9(12):e113649. doi: 10.1371/journal.pone.0113649 (PMC4249963; doi:10.1371/journal.pone.0113649)
Supplement: Table S1 — q-PCR primer list used in the study. (PDF) [file pone.0113649.s005.pdf]

Name	Sequence 5'—3'			
18s-f	CGAACGTCTGCCCTATCAACT			
18s-r	CAGACTTGCCCTCCAATGGATCCTCGTT			
RPL7-f	ATCTACAAGCGTGGTTATG			
RPL7-r	TGCCGTATTTACCAAGAG			
BRI3-f	CAGGGATACCCACCCACCA			
BRI3-r	CAGCCTCCTACGACCACGATA			
SMOC1-f	TAACAAGCGGGAGATGAA			
SMOC1-r	CACAGTAGTCGGTGAAACG			
ANKMY1-f	AGATGGCTCCAGTTTCACG			
ANKMY1-r	CGCTGGTCCGCTTTGTAT			
ATP2C1-f	TTGGTTCCAGGTGATACAG			
ATP2C1-r	AGCCTCAAACAAGCGTAA			
COL14A1-f	TGGACAGAGGAAATGGGAGT			
COL14A1-r	GACCAGGATTACAATGTCAGCA			
DLG1-f	AGCCTTAGCCCTAGTGTA			
DLG1-r	CTCTTGAGGAGGTGTATCT			
KCNC4-f	GGCACGACATGGCATCATAC			
KCNC4-r	GTTGCCTGACCAACTTGAGC			
