# Supplementary material for: Nifedipine Promotes the Proliferation and Migration of Breast Cancer Cells
Source: PLoS One. 2014 Dec 1;9(12):e113649. doi: 10.1371/journal.pone.0113649 (PMC4249963; doi:10.1371/journal.pone.0113649)
Supplement: Table S2 — Up-regulated genes from nude mice tumors with the treatment of nifedipine compared with CMC-Na. (PDF) [file pone.0113649.s006.pdf]

Gene ID	Gene_symbol	Gene Description	log2 (Ratio) N/C	P-value N/C
PH_hs_0000323	MMP14	matrix metallopeptidase 14 (membrane-inserted)	0.60727	0.005601
PH_hs_0001350	SMOC1	SPARC related modular calcium binding 1	1.213168	0.015192
PH_hs_0002197	NCLN	nicalin	0.831504	0.013954
PH_hs_0002246	TMEM39B	transmembrane protein 39B	0.630961	0.043925
PH_hs_0003267	TMEM35	transmembrane protein 35	1.216831	0.002852
PH_hs_0003866	VWCE	von Willebrand factor C and EGF domains	0.837565	0.004939
PH_hs_0005216	LLGL1	lethal giant larvae homolog 1 (Drosophila)	0.904812	0.036394
PH_hs_0006636	DDN	dendrin	0.708493	0.026401
PH_hs_0006992	KCNC4	potassium voltage-gated channel, Shaw-related subfamily, member 4	0.83928	0.000258
PH_hs_0008728	PART1	prostate androgen-regulated transcript 1 (non-protein coding)	0.926475	0.001214
PH_hs_0013578	COPG2	coatomer protein complex, subunit gamma 2	0.787935	0.034171
PH_hs_0013741	APOC1	apolipoprotein C-I	0.755649	0.00955
PH_hs_0016057	TRIM3	tripartite motif containing 3	0.900484	0.006867
PH_hs_0017841	LOC100505903	uncharacterized LOC100505903	0.828699	0.00842
PH_hs_0017903	LOC100507086	uncharacterized LOC100507086	1.164524	0.049071
PH_hs_0019132	COL14A1	collagen, type XIV, alpha 1	0.847235	0.012455
PH_hs_0021501	LOC100506503	uncharacterized LOC100506503	0.915449	0.010098
PH_hs_0023691	LOC283050	uncharacterized LOC283050	0.963049	0.012038
PH_hs_0025376	ANKMY1	ankyrin repeat and MYND domain containing 1	0.716278	0.020398
PH_hs_0025732	RCN3	reticulocalbin 3, EF-hand calcium binding domain	1.122222	0.000001
PH_hs_0027188	NPM3	nucleophosmin/nucleoplasmin 3	1.148084	8.38E-09
PH_hs_0027196	ASIC3	acid-sensing (proton-gated) ion channel 3	0.970299	0.000008
PH_hs_0027321	TAAR5	trace amine associated receptor 5	0.753434	0.016555
PH_hs_0028562	PDE4DIP	phosphodiesterase 4D interacting protein	0.585784	0.019663
PH_hs_0029642	MRPL9	mitochondrial ribosomal protein L9	0.682428	0.002337
PH_hs_0031317	TUBA4A	tubulin, alpha 4a	0.699798	0.00692
PH_hs_0031633	MGC34034	uncharacterized protein MGC34034	0.618797	0.017446
PH_hs_0032018	IRF2BPL	interferon regulatory factor 2 binding protein-like	0.763812	0.018973
PH_hs_0032323	SUPT6H	suppressor of Ty 6 homolog (S. cerevisiae)	0.870252	0.00018
PH_hs_0033589	SLC22A31	solute carrier family 22, member 31	0.697127	0.005816
PH_hs_0035885	KCNS2	potassium voltage-gated channel, delayed-rectifier, subfamily S, member 2	1.040915	0.000882
PH_hs_0037329	LOC100616668	TPTE2 pseudogene	1.038196	0.015472
PH_hs_0039037	STAG3L2|STAG3L1|STAG3L3	stromal antigen 3-like 2|stromal antigen 3-like 1|stromal antigen 3-like 3	0.738154	0.00128
PH_hs_0039928	SNHG5	small nucleolar RNA host gene 5 (non-protein coding)	0.782736	0.020896
PH_hs_0042511	BRI3	brain protein I3	1.098499	0.008855
PH_hs_0043346	MMS22L	MMS22-like, DNA repair protein	0.679814	0.000743
PH_hs_0043935	NPSR1	neuropeptide S receptor 1	0.960309	0.027762
PH_hs_0044076	LOC100129831	EPWW6493	0.765508	0.000327
PH_hs_0044148	TNNI1	troponin I type 1 (skeletal, slow)	0.651846	0.016463
PH_hs_0044637	QSOX1	quiescin Q6 sulfhydryl oxidase 1	1.006846	0.000998
PH_hs_0044911	OR10C1	olfactory receptor, family 10, subfamily C, member 1	0.875152	0.035573
PH_hs_0046008	SIGLEC7	sialic acid binding Ig-like lectin 7	0.742082	0.04941
PH_hs_0048251	RNF187	ring finger protein 187	1.072906	0.029651
**N/C represents the mRNA abundance ratio between nifedipine groups and CMC-Na groups.**				
