# Supplementary material for: Nifedipine Promotes the Proliferation and Migration of Breast Cancer Cells
Source: PLoS One. 2014 Dec 1;9(12):e113649. doi: 10.1371/journal.pone.0113649 (PMC4249963; doi:10.1371/journal.pone.0113649)
Supplement: Table S4 — 35 genes related to cell adhension and migration of all the 69 changed genes. (PDF) [file pone.0113649.s008.pdf]

PH_ID	Gene Symbol	log2 (Ratio) N/C	P-value N/C
PH_hs_0019132	COL14A1	0.847235	0.012455
PH_hs_0044690	ATP2C1	-0.660125	0.005659
PH_hs_0031317	TUBA4A	0.699798	0.00692
PH_hs_0000289	HIST2H2BE	-0.762272	0.035867
PH_hs_0031085	RACGAP1	-0.674306	0.044193
PH_hs_0028562	PDE4DIP	0.585784	0.019663
PH_hs_0001350	SMOC1	1.213168	0.015192
PH_hs_0006992	KCNC4	0.83928	0.000258
PH_hs_0041090	SLC2A3	-1.384045	0.007048
PH_hs_0044148	TNNI1	0.651846	0.016463
PH_hs_0000323	MMP14	0.60727	0.005601
PH_hs_0003866	VWCE	0.837565	0.004939
PH_hs_0023616	RINT1	-0.764899	0.01971
PH_hs_0046008	SIGLEC7	0.742082	0.04941
PH_hs_0044637	QSOX1	1.006846	0.000998
PH_hs_0040856	ADAM9	-1.226181	0.000038
PH_hs_0005216	LLGL1	0.904812	0.036394
PH_hs_0029642	MRPL9	0.682428	0.002337
PH_hs_0024513	DLG1	-0.825158	0.039894
PH_hs_0006636	DDN	0.708493	0.026401
PH_hs_0035885	KCNS2	1.040915	0.000882
PH_hs_0002246	TMEM39B	0.630961	0.043925
PH_hs_0013741	APOC1	0.755649	0.00955
PH_hs_0026484	TMEM170A	-0.696725	0.03891
PH_hs_0044911	OR10C1	0.875152	0.035573
PH_hs_0027188	NPM3	1.148084	8.38E-09
PH_hs_0002197	NCLN	0.831504	0.013954
PH_hs_0043935	NPSR1	0.960309	0.027762
PH_hs_0025234	OXR1	-0.672135	0.026326
PH_hs_0013578	COPG2	0.787935	0.034171
PH_hs_0025732	RCN3	1.122222	0.000001
PH_hs_0003267	TMEM35	1.216831	0.002852
PH_hs_0027321	TAAR5	0.753434	0.016555
PH_hs_0002508	SSR3	-0.666408	0.020462
PH_hs_0042511	BRI3	1.098499	0.008855
